# Factors That Influence Participation in Physical Activity in School-Aged Children and Adolescents: A Systematic Review from the Social Ecological Model Perspective

**DOI:** 10.3390/ijerph18063147

**Published:** 2021-03-18

**Authors:** Donglin Hu, Shi Zhou, Zachary J. Crowley-McHattan, Zhiyun Liu

**Affiliations:** 1 Faculty of Health, Southern Cross University, Lismore, NSW 2480, Australia; Shi.Zhou@scu.edu.au (S.Z.); Zac.Crowley@scu.edu.au (Z.J.C.-M.); 2 School of Physical Education and Educational Science, Tianjin University of Sport, Tianjin 301617, China; 3 Department of Physical Education, Nanjing Agricultural University, Nanjing 210095, China

**Keywords:** physical activity, children and adolescents, social ecological model, participation in sport and exercise

## Abstract

High prevalence of physical inactivity and obesity in children and adolescents has become a global problem. This systematic review aimed to examine the existing literature regarding the factors that influence participation in physical activity (PA) in children and adolescents with reference to the social ecological model (SEM) proposed by McLeroy et al. (1988). The SEM provides a framework under which the influencing factors are categorized into five levels: intrapersonal, interpersonal, organizational, community, and public policy. A systematic search of relevant literature published before July 2020 was conducted through Ebsco, ProQuest, PubMed Central, Scopus, and Web of Science. A total of fourteen studies met the inclusion criteria. The selected articles were all of high quality as assessed using the Mixed Methods Appraisal Tool (2018). The results indicated that gender, age, ethnicity, and self-concept were the most common influencing factors at the intrapersonal level. At the interpersonal and organization levels, supports from friends, parents, and teachers were positive predictors of students’ PA participation. Accessibility of facilities and safe neighborhoods was a crucial factor that influenced children and adolescents’ participation in PA at the community level. Future studies on the effective types of policies or practices that could successfully promote facilities’ accessibility and improve neighborhood safety are required. The outcomes of this systematic review are expected to inform practice and support the development and implementation of sound policies for the promotion of PA participation in children or adolescents from a comprehensive social ecological viewpoint.

## 1. Introduction

Physical activity (PA) refers to any bodily movement produced by skeletal muscles that requires energy expenditure [1,2]. Participation in regular and adequate levels of PA is an essential contributor to good health, maintenance of healthy weight, and management of risk factors of chronic diseases [3,4]. However, the current PA participation levels in developed countries are generally less than the optimal level recommended to gain health benefits in both adults and children [1,2]. There is little doubt that participation in PA is inversely related to being overweight and the risks of metabolic and cardiovascular diseases, at least as found in cross-sectional studies [5]. There is strong evidence that participating in the recommended amount of PA is beneficial to children and adolescents, improving physical and mental health, sleep quality, brain development, bone health, and social, psychological, and cognitive health [6]. Furthermore, existing evidence shows that people’s behavior in adulthood stems from the environment they have lived in since childhood, and that the behavioral habits developed in childhood tend to sustain in adulthood [7].

Inadequate PA levels and increased prevalence of obesity in children and adolescents has become a global issue [1]. Although PA is an essential component of health interventions, various intrapersonal factors and environmental barriers may prevent children and adolescents from participating in adequate levels of PA [8]. Some researchers have previously tried to identify and understand factors leading to inadequate PA levels in children or adolescents, but they primarily focused on the factors at the individual level such as self-efficacy [9,10,11]. A growing body of research, based on social determinants of health perspectives, demonstrates that engaging in health enhancing behaviors such as participating in PA is far from being only a matter of an individual’s decision or intention but also influenced by the social and physical environments [12,13]. To identify the critical factors that influence people’s level of participation in PA and understand the relationships between these factors, the application of a social ecological model (SEM) as an organizational framework has been advocated by many researchers [14,15,16,17].

## 2. The Social Ecological Model

**Figure 1:**
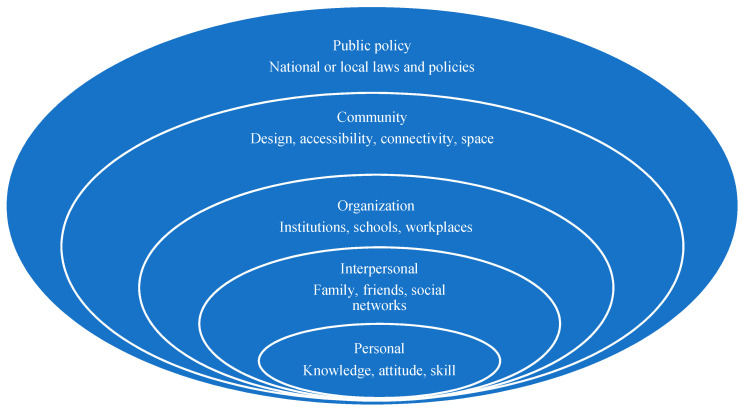
The social ecological model adapted from McLeroy, K.R., Bibeau, D., Steckler, A., and Glanz, K. (1988) [16]. An ecological perspective on health promotion programs.

Engaging in PA is a complex issue because exercise-related behavior is multifaceted and affected by many factors to varying degrees [18]. Both personal and social environmental factors can contribute to behavioral changes [16]. To address this complex problem, an SEM was developed [16] that has become a useful tool for exploring the multiple factors involved in PA participation rates and adherence in children and adolescents [19]. The SEM suggests that the PA behavior is determined or affected by the following five levels or groups of factors: (1) intrapersonal factors; (2) interpersonal processes; (3) organizational factors; (4) community factors; and (5) public policy. In addition to clarifying the specific effects of different levels on health behavior, McLeroy et al. (1988) [16] described the possible interventional strategies at varying levels of impact (see Figure 1) and suggested that interventions (1) at the intrapersonal level aim to change an individual’s knowledge, attitudes, behavior, self-concept, or skills, etc.; (2) at the interpersonal level aim to address formal and informal social networks and social support systems, including family, work groups, and friendship networks; (3) at the organization level can identify factors concerning the school, workplace, or university and may also include influences from teachers and school administrators; (4) at the community level involve modifying the community environment or services and the relationships among organizations; and finally (5) at the public policy level involve the creation or modification of public policies, including local, state, and national laws and policies (Figure 1).

The SEM describes that an individual is embedded in a social system, and the interactive characteristics of the individual and the environment form the basis of health outcomes [20]. The SEM is based on the assumption that the combination of individual, social, and physical environmental factors will best explain PA participation [17]. Given that PA must take place in a particular physical environment that may affect an individual’s choice to engage in PA, the SEM is particularly appropriate for studying PA. Application of the SEM can help improve PA participation by examining the intrapersonal (e.g., gender, age, self-concept), social environmental (parents, teachers, friends), and physical environmental (safety, facility, and space accessibility) factors that may influence one’s decision to participate in PA at an adequate level [21].

Many reports and reviews in the literature have examined the factors that influence PA or sports participation in children and adolescents [22,23,24,25,26,27,28,29,30,31,32,33,34,35,36,37]. However, although these previous works have studied or reviewed some of the factors affecting children and adolescents’ participation in PA, none of them have comprehensively examined the factors with reference to all five levels in the SEM established by McLeroy et al. [16]. Although different social ecological models may have their limitations, the SEM by McLeroy et al. (1988) is unique insofar as it delineates between institutional and community levels of influence. Within the context of children and adolescents’ PA, research and practice typically occur within these two levels/sectors (i.e., institution/school-based exercise and community-based sport and exercise). Therefore, this systematic review was based on McLeroy et al.’s SEM perspective. This systematic review aimed to address knowledge gaps in the literature through (1) identifying and synthesizing findings from the current literature that have explored factors affecting the participation of children and adolescents in the construct of the SEM and (2) assessing the quality of the studies that applied the framework of the SEM. In the present review, the quality appraisal was performed using the Mixed Methods Appraisal Tool (MMAT) [38]. This systematic review’s outcomes are expected to inform the practice and support the development and implementation of sound practice and policies for the promotion of PA participation in children or adolescents from a comprehensive social ecological viewpoint.

## 3. Methods

**Figure 2:**
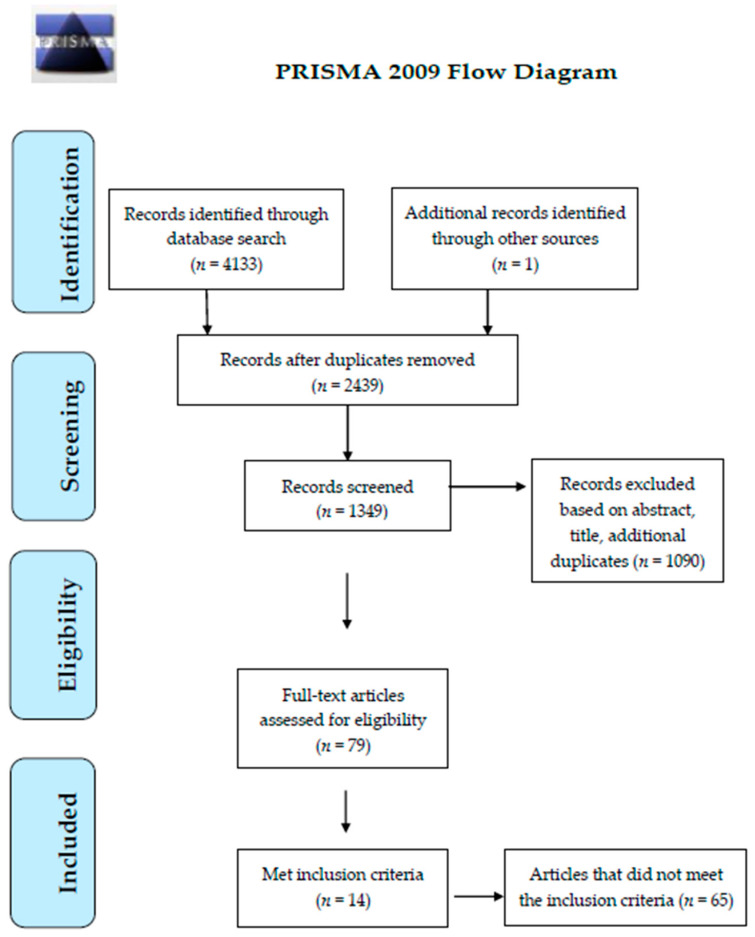
Preferred Reporting Items for Systematic Reviews and Meta-Analyses (PRISMA) flow diagram.

A search of the literature was conducted on 19 June 2020 through the following electronic databases: EBSCO (including AMED, CINAHL Plus, Health Business, Health Source -Nursing/Academic Edition, MEDLINE with Full Text, APA PsycArticles, Psychology and Behavioral Sciences Collection, APA PsycInfo, and SportDiscus), ProQuest, PubMed Central (PMC), Scopus, and Web of Science. Searches were limited to articles published in the English language. The time range was set to all years, as it was both feasible and comprehensive, and in this way, the maximum numbers of the articles would be included. A Boolean search strategy was used to identify articles that had a combination of the following keywords: (“socio-ecological model” or “social ecological model” or “social ecological theory”) AND (“physical activity” or “exercise or fitness” or “physical exercise” or “sport”) AND (children or adolescents or youth or child or teenager) (see Table A1 in Appendix for more detailed search setting). Search outcomes were reported according to the Preferred Reporting Items for Systematic Reviews and Meta-Analyses (PRISMA) guidelines [39]. Figure 2 shows the PRISMA diagram of the article screening process.

The following inclusion and exclusion criteria were used to identify the eligible articles for review, and only empirical research articles were considered. 

Inclusion criteria: (1) full-text available; (2) the research participants were healthy children or adolescents; (3) the research made a reference to the SEM or social ecological theory; (4) written in English; and (5) published in scholarly (peer reviewed) journals. 

Exclusion criteria: (6) books, book sections, dissertations, thesis, or conference abstracts; (7) studies on participants of preschool age or younger (i.e., under 7 years of age); (8) studies focused on nutritional interventions or healthy eating; (9) studies focused on sedentary behavior only; (10) studies focused on active transportation; (11) studies focused on disabled and overweight populations only; and (12) studies focused on the influence of (electronic device) screen time.

## 4. Data Extraction

Two researchers (D.H. and S.Z.) searched the databases and assessed the articles’ titles and abstracts separately to determine the initial inclusions. If discrepancies were found and could not be resolved between the two researchers, a third researcher (Z.C.M.) was engaged to finalize the assessment. The full texts were then assessed against the inclusion and exclusion criteria to finalize the articles eligible for inclusion in the review. The information extracted from the full text included methodological, demographic, and outcome data, including reported children/adolescents’ characteristics (number of participants, participants’ age range, gender), the location of studies, levels of SEM applied, research methods, and results. Text units (a unit refers to a sentence or paragraph that represents one idea) regarding the influencing factors on PA participation were identified and labelled as either a “barrier” or “facilitator”. For ease of presentation, the symbols of “+” and ‘–’ were used for facilitators and barriers, respectively. In many cases, an article explored both barriers and facilitators for each factor (e.g., “the support of teachers” is a facilitator whereas “lack of support from teachers” is a barrier). In such a case, we used ‘+–’ to capture all of these factors together. In addition, if there were no significant association reported between some factors and PA participation in an article, we used “0” to represent the result. Although a few articles mentioned some factors, they did not report the relationship between these factors and PA. In such cases, we use the abbreviation “NR” for “not reported”. Discrepancies between researchers were resolved through interactive discussions. Table 1 delineates the main characteristics of these studies.

## 5. Results

A total of 4134 articles were identified in the search process (Figure 2). Fourteen articles met the inclusion criteria (Table 2). Among these articles, three studies were conducted in Australia, two in each of the United States, Spain, and Canada, and one in each of Denmark, Japan, the United Kingdom, Morocco, and Israel. Among the fourteen articles, there were seven qualitative and seven quantitative studies. In the qualitative studies, five of the seven studies adopted a focus group method. Except for two qualitative studies that interviewed adults [40,41], all the study participants were children and adolescents. In the quantitative research, a self-report questionnaire survey was mostly used, with six of the seven studies adopting this method. As shown in Table 2, concerning the five levels of the SEM, most studies focused on three or four levels, while four articles addressed four levels of the SEM, eight articles addressed three levels, two articles addressed two levels, and none of them addressed all five levels.

Our analysis revealed that 12 articles addressed the first four levels, while only two addressed the fifth level—policy. In addition, most (10) articles [8,40,41,42,43,44,45,46,47,48] examined school-based PA, indicating that schools were the most common setting for children and adolescents to participate in PA. In line with previous reports [49,50,51], the school was identified as the primary location of organized PA for children and adolescents.

Table 1 categorizes the factors that influence PA participation regarding the SEM [16]. At the interpersonal level, there were many facilitators (25), while at the community level, barriers (23) were the most prevalent. There were 19 intrapersonal, 17 interpersonal, 10 organizational, and 23 community level (69 in total) barriers, and 15 intrapersonal, 25 interpersonal, 15 organizational, 9 community, and 4 policy level (68 in total) facilitators.

**Table 1:** The factors that influence participation in physical activity in children and adolescents in the social ecological model proposed by McLeroy et al. (1988) [16].

Level Description of Factors		Study Reference Number														Total Number of			
		1	2	3	4	5	6	7	8	9	10	11	12	13	14	+	−	0	NR
Intrapersonal																15	19	13	
	Self-concept		+−	+−	+−			+−	+−						+−	6	6		
	Alcohol			0														1	
	Smoking			−											−		2		
	Mental health						0											1	
	Pediatric Quality of Life Inventory (Physical, Emotional, Social, School Functioning)						0											1	
	Temperament						0											1	
	Time in PA						0											1	
	Levels of PA						0											1	
	Age, years			0					+−	+−				+−		3	3	1	
	Race/ethnicity								0		−	−		0			2	2	
	Gender						+−		0	+−	+−	+−		+−	+−	6	6	1	
	BMI			0			0		0									3	
Interpersonal																25	17	6	1
	Friends’ influence		+	+−	+−	+−		+−	+	+		+		+	+−	10	5		
	Parents’ influence		+	+−	+−					+				+	+−	6	3		
	Parents’ employment status										+−					1	1		
	Conflicts					−											1		
	Speak English as a main language						+									1			
	Fewer people in family						+				−					1	1		
	Parent education						+−		+−					+−		3	3		
	Household economic state						+−			NR	−					1	2		1
	Parental concern about child’s weight						0											1	
	Parental PA with child						0							0				2	
	Parental PA			+−			0							0		1	1	2	
	Child taken to sporting event						+									1			
	Parenting style						0											1	
Organization																15	10	1	
	School culture support	+											+			2			
	Principals’ support	+											+			2			
	Teachers’ influence	+	+			−	+	+−					+			5	2		
	Good PE grade			+												1			
	Type of school			+−						+−						2	2		
	Designing enjoyable class experiences			+												1			
	School management and arrangement					−		−				+			+−	2	3		
	School safety							−									1		
	Child gets bullied at school						0	−									1	1	
	Time constraints							−									1		
Community																9	23	5	
	Facilities accessibility		+			−		+−	+−		−				+−	4	5		
	Availability of space					−	0	+−			−					1	3	1	
	Neighborhood safety		0		−		0		0	−	−						3	3	
	Distance				+−				+−					0		2	2	1	
	Weather				+−	−		+−		−						2	4		
	Rural aeras										−						1		
	Lack of time				−					−							2		
	Active transportation										−						1		
	Use of electronic devices					−				−							2		
Policy																4			
	School board policy	+														1			
	Provincial government policies	+														1			
	Municipal government policies	+											+			2			

BMI: body mass index. LTPA: leisure time physical activity. PA: physical activity. PE: physical education. +: facilitator. −: barrier. 0: no significance. NR: not report. 1 = Langille and Rodgers (2010) [40], 2 = Zhang et al. (2012) [42], 3 = Bengoechea et al. (2013) [52], 4 = Stanley et al. (2013) [48], 5 = Pawlowski et al. (2014) [43], 6 = Vella et al. (2014) [53], 7 = Stanley et al. (2012) [47],8 = D’Angelo et al. (2017) [46], 9 = Martinez-Andres et al. (2020) [54], 10 = Taylor et al. (2018) [55], 11 = Tesler et al. (2019) [44], 12 = Webster et al. (2014) [41], 13 = Wilk et al. (2017) [45], 14 = El-Ammari et al. (2019) [8].

**Table 2:** Summary of included qualitative and quantitative studies reporting the factors that influence participation in physical activity in children and adolescents.

Article ID	Number of Participants	Age (Years)	Sex	Study Location	Sample Selection	Levels of SEM	Collection Method	Instrument	Type of Study	Physical Activity Periods
1	*n* = 14 Members of the Government, Public-School Board (PSB), Principals and Teachers.	NI	8 females 6 males	Canada	Intentional	Organization Policy	In-depth interview	Convenience and snowball sampling A conversational structure Interviews	Qualitative	School-based PA
2	*n* = 285	Aged 12–15 years	Boys = 142 Girls = 143	Middle school, Southern state, USA	Intentional	Intrapersonal Interpersonal Organization Community	Questionnaire	PAQ-C Questionnaires Motl et al.	Quantitative	School-based PA
3	*n* = 3249	Aged 12–17 years	1548 females 1701 males	Southeastern Spain	Intentional	Intrapersonal Interpersonal Organization	Questionnaire	Question, Canadian Institute for Health Information. Improving the health of young Canadians	Quantitative	After-school Leisure-time PA
4	*n* = 54	Aged 10–13 years	Girls = 31 Boys = 23	South Australia	Intentional	Intrapersonal Interpersonal Community	Focus groups	Focus groups Question A semi-structured questioning route	Qualitative	After-school (3:30–6:00 PM) PA
5	*n* = 111	Aged 10–11 years	Boys = 53 Girls = 58	Denmark	Intentional	Interpersonal Organization Community	Focus groups	Focus group, discussion, interviews, and a gender segregated post-it note activity	Qualitative	School recess PA
6	*n* = 4164	Aged 8–9 and 10–11 years	Boys = 2069 Girls = 2095	Australia	Random	Intrapersonal Interpersonal Organization Community	Questionnaire	Longitudinal Study of Australian Children (LSAC), Question, Questionnaires	Quantitative	Organized sports
7	*n* = 54	Aged 10–13 years	Girls = 31 Boys = 23	South Australia	Intentional	Intrapersonal Interpersonal Organization Community	Focus groups	Focus groups Question A semi-structured questioning route	Qualitative	School based lunchtime PA
8	*n* = 1263	Aged 12–17 years	637 females 626 males	USA	Intentional	Intrapersonal Interpersonal Community	Questionnaire	Questionnaires The self-reported Youth Activity Profile (YAP)	Quantitative	School and out of school section PA
9	*n* = 98	Aged 8–11 years	NI	Cuenca, Spain	Intentional	Intrapersonal Interpersonal Community	Focus groups	Analysis of the children’s drawings of their environment focus groups questions	Qualitative	After-school PA
10	*n* = 892	Aged 8–14 years	Boys = 396 Girls = 496	Ontario, Canada	Random	Intrapersonal Interpersonal Community	Questionnaire	Question measuring barrier Questionnaires	Quantitative	Out of school section PA
11	*n* = 16,145	Grades = 6, 8, 10, 11, and 12	Boys = 7764 Girls = 8381	Israel	Random	Intrapersonal Interpersonal Organization	Questionnaire	2014–15 Health Behavior of School-Aged Children standardized survey. Self-reported questionnaires	Quantitative	School and out of school section PA
12	Classroom teachers/PE program leaders, principals, district officials, and a Ministry of Education official	NI	NI	Japan	Intentional	Organization Policy	Interview	Semi-structured interviews, Observation data (field notes, photographs, and videos)	Qualitative	School-based PA
13	*n* = 957 children *n* = 1440 parents	Aged 9–11 years, children	Boys = 459 Girls = 456	London, England	Random	Intrapersonal Interpersonal Community	Questionnaire	The Grade 5 ACT-i-Pass (G5AP) the 2014–15 school year Child and parent questionnaires	Quantitative	School and out of school section PA
14	*n* = 56 adolescents *n* = 26 parents *n* = 18 teachers	Aged 14–16 years Others aged 30–60 years	Boys = 28 Girls = 28	Taza, Morocco	Random	Intrapersonal Interpersonal Organization Community	Focus groups	Semi-structured interviews	Qualitative	School and out of school section PA

NI: not informed. PA: physical activity. 1 = Langille and Rodgers (2010) [40], 2 = Zhang et al. (2012) [42], 3 = Bengoechea et al. (2013) [52], 4 = Stanley et al. (2013) [48], 5 = Pawlowski et al. (2014) [43], 6 = Vella et al. (2014) [53], 7 = Stanley et al. (2012) [47],8 = D’Angelo et al. (2017) [46], 9 = Martinez-Andres et al. (2020) [54], 10 = Taylor et al. (2018) [55], 11 = Tesler et al. (2019) [44], 12 = Webster et al. (2014) [41], 13 = Wilk et al. (2017) [45], 14 = El-Ammari et al. (2019) [8].

## 6. Quality Appraisal

Articles that met the selection criteria were critically assessed for their quality according to the MMAT 2018, including the soundness of the methods and the extent to which there might be bias in the research design, how the research was conducted, and data analysis techniques [56]. The MMAT is designed to evaluate mixed studies (i.e., utilized qualitative, quantitative, or mixed method). The MMAT has two screening questions for different types of studies, and there are five questions for each of the two possible research design types to assess the quality of the research. Table 3 shows the results from the quality evaluation. Eight of the fourteen articles eligible for review reached positive ratings for all questions. At the same time, no study had more than one negative rating, indicating that the selected articles were all of high quality.

## 7. Discussion

This systematic review aimed to examine the empirical research from the existing literature regarding the factors that influence PA participation in children and adolescents through the lens of the SEM established by McLeroy et al. (1988). In general, only a small number of studies (14) met the inclusion criteria. None of these studies addressed the factors at all five levels in the SEM, and only two studies addressed the factors at the policy level. As analyzed by the MMAT [38], the selected studies’ quality analysis showed that the articles that met the inclusion criteria could be regarded as high quality (Table 3).

Among the seven quantitative studies, five had large sample sizes of more than 1000 [44,45,46,52,53] and utilized self-report questionnaires, while none of these studies utilized actual measurements of PA (Table 2). Meanwhile, none of these quantitative studies addressed the policy level factors in all the questionnaires adopted. Future research should consider adopting more objective assessments, such as accelerometers and heart rate monitors or other valid methods, to investigate students’ actual PA. In addition, a new questionnaire with policy factors could also be considered.

Compared with the quantitative studies, the selected qualitative studies had a relatively smaller sample size of around 100 (Table 2). A smaller sample size was convenient for focus group or interview research methods. Regarding the methodology employed within the studies reviewed, the focus group was the most utilized in the qualitative studies [8,43,47,48,54]. The focus group method is well suited to qualitative research on children and adolescents because children communicate more readily with their peers than adults [57]. Two of the qualitative studies addressed the factors at the policy level [40,41]. The two studies adopted the interview approach. The interview approach was deemed appropriate to answer the research questions, as it allowed researchers to explore the interesting issues in depth [41]. Qualitatively exploring policy factors that influence PA participation can be an important step in questionnaire development [48]. Future research could consider designing policy factors obtained from qualitative studies into questionnaire development. 

This systematic review focused on influencing factors identified in the literature at each level of the SEM. The highest number of barriers were found at the community level, while the number of facilitators was highest within the interpersonal level.

Intrapersonal level: The most frequently cited factors at the intrapersonal level were gender, self-concept, age, ethnicity, and body mass index (BMI). Eleven studies examined these factors and their relationships to PA participation [8,42,44,45,46,47,48,52,53,54,55]. When it came to gender and age, most studies were consistent. Six studies indicated that boys were more active than girls, and boys spent more time on recreational PA [8,44,45,53,54,55]. These gender differences were explained by non-modifiable variables, including girls’ biology [58], and by some modifiable variables such as psychological [59] and cultural background factors [60]. In addition, older children were found to be less active than younger children, so in childhood or adolescence, older age could be viewed as a barrier, while younger ages may be considered a facilitator [45,46,54]. This finding had also been demonstrated elsewhere [61]. There was a trend that older children, both boys and girls, preferred playing video games at home and watching TV compared to playing physical games in their leisure time [54]. Several questions can be raised for future studies, e.g., what types of age- and gender-appropriate physical activities are attractive to children and adolescents? How to increase opportunities and the likelihood of children and adolescents participating in PA, taking into account gender and age differences? The answer to these questions will help inform school policy and develop strategies designed to promote PA in school settings.

Self-concept and BMI were additionally reported as influencing factors at the intrapersonal level. Self-concept includes self-efficacy [42,46], perceived health, physical self-perception, participation motives [52], and perceived competence and enjoyment [48]. In these studies, self-concept has been one of the strongest predictors of PA participation in children or adolescents [42]. Consistent with previous studies, when children or adolescents have high levels of self-concept, they tend to persist and actively participate in PA, and vice versa [11,62]. This finding suggested that physical education educators and health promoters should aim to improve students’ self-concept continuously and at the same time encourage them to adopt and maintain regular PA. Three studies [46,52,53] showed that BMI was not associated with PA participation. Considering the rate of PA participation in overweight and obese children was similar to that in their normal weight peers [63], these children all might have participated in PA to improve their health. Thus, BMI was not a predictor of PA participation. The directionality of relationships between participation in the PA and measures of physical health still needs more research [1]. Furthermore, two studies [44,55] found that children and adolescents of different ethnic groups had different PA participation levels. Children from visible minority groups were more likely to report more PA barriers than Caucasian children in a study from Canada [55]. Another study from Israel reported that PA participation was different between adolescents from different ethnic backgrounds (Jews and Arabs), which could lead to health disparities [44]. This is in line with the other studies that have found that the differences in PA levels were associated with the ethnic backgrounds in adolescents [64,65]. Therefore, it is suggested that “race/ethnicity” and/or cultural backgrounds should be a consideration in the design of future studies investigating factors that influence PA participation. 

Interpersonal level: The most mentioned factor at the interpersonal level was friends’ influence. There was consistent evidence across the articles regarding the importance of supports from friends and parents in facilitating PA participation [8,42,43,44,45,46,47,48,52,54]. Additionally, a lack of supports from friends or parents was considered a barrier to PA participation for children or adolescents [8,43,47,48,52]. These findings were consistent with previous research [66,67,68], which suggested that supports from parents and friends could promote regular PA participation among children and adolescents and help them develop and maintain an active lifestyle [42]. Furthermore, it appeared that parents’ educational level may have an additional influence on children’s PA participation profile. In Vella, Cliff and Okely [53], lower educational attainment of the parents was identified as a barrier, while D’Angelo, Fowler, Nebeling and Oh [46] and Wilk, Clark, Maltby, Smith, Tucker and Gilliland [45] reported that students whose parents had a college degree or higher levels of education had a moderate to vigorous PA profile. This observation, however, is based on a limited number of studies. Therefore, it is prudent that further investigations are required to investigate the relationship between parents’ education level and children’s PA participation level.

Organization level: Six studies [40,41,42,43,47,53] examined the relationship between teachers’ influence and children’s PA participation. Teachers’ support was a significant positive predictor of PA participation. Five articles found that support from physical education (PE) teachers could positively promote students’ engagement in PA [40,41,42,47,53], and two articles indicated that a lack of teachers’ support was a barrier [43,47], which is consistent with previous studies [69,70]. For example, professional PE teachers in primary schools were shown to be able to improve PA levels and fundamental movement skills better compared to untrained teachers [53,71]. This systematic review also found that different types of schools were associated with children’s PA participation. Private schools and rural schools appeared to positively promote students’ engagement in PA, whereas urban public schools lacked this positive influence. Two studies [52,54] found that boys attending public schools were reportedly participating less in leisure time PA than boys in private schools. In addition, children in rural areas had more leisure time, which was consistent with a previous report [72]. Future studies should explore the reasons for such a difference in PA participation between urban and rural schools and between public and private schools.

Community level: From the analysis of the included studies, this systematic review yielded evidence of the importance of neighborhood safety and accessibility to facilities on PA profiles at the community level. Although three studies [42,46,53] reported that neighborhood safety had no significant effect on PA participation, these samples were predominantly from parents with higher levels of education or from communities with a dominant ethnic group (e.g., Caucasian). Therefore, in future studies it may be prudent to consider other potential influencing factors (e.g., intrapersonal) when investigating the community level. Another three studies [48,54,55] showed that a lack of safety was a significant barrier to PA participation, which was consistent with previous studies [73,74,75]. The discrepancies between studies may be due to differences in settings. Furthermore, facility accessibility was found to be an important factor for students’ positive engagement in PA [8,42,43,46,47,55]. Physical educators and health promoters should advocate the needs of accessible facilities at affordable levels to various participants in the community to promote PA participation [76]. However, building safer neighborhoods and providing more accessible facilities within the community are often beyond the physical educators’ and health promoters’ capacity. Therefore, changes must occur at the policy level. In addition, most school children mentioned that weather was also an important influencing factor [43,47,48,54]. Therefore, it may be beneficial for physical educators to provide children with information on alternative activities to keep them physically active (e.g., adapted skating in winter, indoor PA games on rainy days) when the weather is not promising.

Other factors at the community level that were mentioned but at a much lower frequency were a lack of time and the use of electronic devices [43,48,54]. As children progress to senior years, there was an associated increase in the amount of schoolwork, which might force them to prioritize study activities over other activities, especially sports and PA [48,54]. Additionally, children were more likely to watch TV or play video games in situations where free-play time was limited [43,54]. Strategies to promote PA can focus on balancing competing interests by ensuring that more time is given to PA opportunities, as proposed by Humbert, et al. [77]. Balancing home responsibilities or adjusting school times (e.g., starting and finishing school earlier) to increase PA opportunities were potential solutions suggested by Stanley et al. [48]. Furthermore, Pawlowski et al. (2014) pointed out that electronic devices’ availability and utilization had not previously been identified as a barrier to PA participation in children. Therefore, more research is needed to explore the impact of this relatively new barrier on PA and suggest future directions in this area [78].

Policy level: There was a limited amount of research focused on the policy level, with only two of the fourteen articles included in this review having analyzed this level [40,41]. The possible reason for this lack of research focus was that all the studies reviewed were aimed at children and adolescents in which schools were the most common locations for PA participation [47]. Therefore, most school-based PA studies were concerned with the school environmental factors (e.g., classmates, teachers, PE curriculum, school facilities, etc.), which resulted in examining factors at the SEM’s lower levels. In the two studies that addressed the policy level, Langille and Rodgers [40] indicated that the influence of provincial and municipal policies were consistent with SEM, in that they had a top-down influence on the direction taken by the schools. Provincial policies were to provide guidance for the schools to develop overall standards and achieve specific results. Meanwhile, the policies of the municipal government could indirectly influence the decisions of school administrators. The policy level is of the highest level in the SEM structure, and it has a strong influence on the lower levels within the SEM. It is clear that different policies can simultaneously or independently influence the school environment and children’s participation in PA. In the other study addressing the policy level, Webster, Andrew and Naoki [41] pointed out that when PA policies lacked accountability, schools might be less inclined to implement these policies because of localized factors, such as principals’ and teachers’ beliefs. Webster, Andrew and Naoki [41] also indicated that policy leadership for school PA in the U.S.A. mainly came from the district government where the school was located and to a lesser extent from the state and federal governments. It may also be necessary to increase the role of state government and perhaps even the federal government in generating school PA policies. In addition, there is an important relationship between policy and community levels in the SEM. As mentioned above, building safer neighborhoods and providing more accessible facilities require policy makers to address issues at higher levels. Changes must occur at the policy level. Future studies could explore the policy level influences with more in-depth analysis to help improve PA rates, and when possible, address all five levels together. Moreover, future studies should examine what types of policies or practices can successfully provide accessible facilities and increase neighborhood safety. 

## 8. Limitations

This systematic review focused mainly on the empirical studies that applied the SEM established by McLeroy et al. (1988). The model itself may have some limitations, e.g., not being able to show the relative importance between the levels and factors. There exist other social ecological models or theories [17]. Although we developed and followed a rigorous, systematic protocol, given the ontological and epistemological assumptions inherent to configurative reviews [79], other studies and reviews that followed different SEMs or theories might have addressed the factors differently and might not result in the same conclusions and recommendations. 

## 9. Conclusions

To our knowledge, this is the first study to systematically review the factors that influence participation in PA in children and adolescents from the perspective of the SEM (McLeroy et al., 2018). This review took into consideration the evaluation of the quality of the empirical studies by using the Mixed Methods Appraisal Tool (MMAT 2018). The result showed that the selected articles were all of high quality.

Considerable efforts have been made, as seen in the literature, in compiling the major factors that may affect PA participation in children and adolescents. In this review, these factors were addressed within the framework of the SEM. Based on the comprehensive analysis, the following recommendations have emerged.

(1) Strategies should focus not only on children and adolescents at the intrapersonal level but also on other levels in the SEM and the key stakeholders operating within these levels (e.g., friends, teachers, parents, and school administrators).
(2) At the intrapersonal level, gender was the most commonly reported influencing factor. It is recommended that gender- and age-specific strategies be identified for further interventions to improve PA participation among children and adolescents. Self-concept was the strongest predictor of PA participation in children or adolescents. Therefore, improving students’ self-concept is of great significance in the future.
(3) At the interpersonal and organizational levels, school-based interventions have the potential to improve adolescents’ PA participation rates. Schools are the most common location for children and adolescents to participate in PA and the main location for organized PA. Supports from friends, parents, and teachers are all significant and positive predictors of students’ PA participation. Whether a holistic universal approach or specific approaches tailored to subgroups or individuals is more effective requires further investigation. There is no consistent evidence on the relationship between parents’ education level and children’s PA participation, and therefore this requires further study.
(4) At the community and policy levels, accessibility of facilities (and at affordable level) and safe neighborhoods are crucial to ensuring children and adolescents participate in PA. Health promoters and policy makers should advocate and raise awareness of these needs for their communities. Future studies should examine what types of policies or practices could successfully provide accessible facilities and increase neighborhood safety.

Identifying the factors that influence PA participation can provide policy makers, physical educators, and public health officials with essential information to guide the distribution of initiatives and resources to promote PA and reduce or eliminate health disparities.

## Figures and Tables

**Table 3:** MMAT quality appraisal results.

1. Qualitative						4. Quantitative Descriptive				
	1.1 Is the qualitative approach appropriate to answer the research question?	1.2 Are the qualitative data collection methods adequate to address the research question?	1.3 Are the findings adequately derived from the data?	1.4 Is the interpretation of results sufficiently substantiated by data?	1.5 Is there coherence between qualitative data sources, collection, analysis, and interpretation?	4.1 Is the sampling strategy relevant to address the research question?	4.2 Is the sample representative of the target population?	4.3. Are the measurements appropriate?	4.4 Is the risk of nonresponse bias low?	4.5 Is the statistical analysis appropriate to answer the research question?
1	Y	C	Y	Y	Y					
2						Y	C	Y	N	Y
3						Y	Y	C	N	Y
4	Y	Y	C	C	Y					
5	Y	Y	Y	Y	Y					
6						Y	Y	Y	Y	Y
7	Y	Y	Y	Y	Y					
8						Y	Y	Y	Y	Y
9	Y	Y	Y	Y	Y					
10						Y	Y	Y	Y	Y
11						Y	Y	Y	Y	Y
12	Y	C	Y	Y	Y					
13						Y	Y	Y	C	Y
14	Y	Y	Y	Y	Y					

Y = YES, N = NO, C = Cannot tell; Mixed Methods Appraisal Tool (MMAT), version 2018 [38]. 1. Qualitative 2. Quantitative randomized controlled trials 3. Quantitative nonrandomized 4. Quantitative descriptive 5. Mixed methods. 1 = Langille and Rodgers (2010) [40], 2 = Zhang et al. (2012) [42], 3 = Bengoechea et al. (2013) [52], 4 = Stanley et al. (2013) [48], 5 = Pawlowski et al. (2014) [43], 6 = Vella et al. (2014) [53], 7 = Stanley et al. (2012) [47],8 = D’Angelo et al. (2017) [46], 9 = Martinez-Andres et al. (2020) [54], 10 = Taylor et al. (2018) [55], 11 = Tesler et al. (2019) [44], 12 = Webster et al. (2014) [41], 13 = Wilk et al. (2017) [45], 14 = El-Ammari et al. (2019) [8].

## Data Availability

Not applicable.

## Appendix A

**Table A1:** Literature search strategy.

Databases and Date Range	Search Terms	Specific Limits	Number of Records Found
EBSCO All years (including following databases) TX (ALL TEXT)	(“socio-ecological model” OR “social ecological model” OR “social ecological theory”) AND (“physical activity” OR “exercise” OR “fitness” OR “physical exercise” OR “sport”) AND (“children” OR “adolescents” OR “youth” OR “child” OR “teenager”)	Boolean/Phrase Apply equivalent subjects English	1370
AMED		Document type: Journal Article Language: English	0
CINAHL Plus		Scholarly (Peer Reviewed) Journals English Research Article, Publication type: Journal Article	98
Health Business Elite		Scholarly (Peer Reviewed) Academic Journals English	67
Health Source (Nursing/Academic Edition)		Publication type: Academic Journal, Document type: Article Scholarly (Peer Reviewed) Journals	120
MEDLINE with Full Text		Publication type: Journal Article English Scholarly (Peer Reviewed) Journals	556
APA PsycArticles		Document type: Journal Article Scholarly (Peer Reviewed) Journals	56
Psychology and Behavioral Sciences Collection		Document type: Article Scholarly (Peer Reviewed) Journals	147
APA PsycInfo		Publication type: Peer Reviewed Journals, Scholarly (Peer Reviewed) Journals Document type: Journal Article English	34
Databases and date range	Search terms	Specific limits	Number
SPORTDiscus with Full Text		English Scholarly (Peer Reviewed) Journals Publication type: Academic Journal, Document type: Article	292
ProQuest All years	ft(“socio-ecological model” OR “social ecological model” OR “social ecological theory”) AND ft(“physical activity” OR “exercise” OR “fitness” OR “physical exercise” OR “sport”) AND ft(“children” OR “adolescents” OR “youth” OR “child” OR “teenager”)	Limit to peer reviewed Source type: scholarly journals Document type: article English	1101
PubMed Central All years	((“socio-ecological model” (All Fields) OR “social ecological model” (All Fields) OR “social ecological theory” (All Fields)) AND (“physical activity” (All Fields) OR “exercise” (All Fields) OR “fitness” (All Fields) OR “physical exercise” (All Fields) OR “sport” (All Fields))) AND (“children” (All Fields) OR “adolescents” (All Fields) OR “youth” (All Fields) OR “child” (All Fields) OR “teenager” (All Fields))	“All Fields” for all the rows	1411
SCOPUS All years	(TITLE-ABS-KEY (“socio-ecological model” OR “social ecological model” OR “social ecological theory”) AND TITLE-ABS-KEY (“physical activity” OR “exercise” OR “fitness” OR “physical exercise” OR “sport”) AND TITLE-ABS-KEY (“children” OR “adolescents” OR “youth” OR “child” OR “teenager”)) AND (LIMIT-TO (DOCTYPE, “ar”)) AND (LIMIT-TO (LANGUAGE, “English”)) AND (LIMIT-TO (SRCTYPE, “j”))	Article, Journal English	125
Web of Science Core Collection All years	TOPIC: (“socio-ecological model” OR “social ecological model” OR “social ecological theory”) AND TOPIC: (“physical activity” OR “exercise” OR “fitness” OR “physical exercise” OR “sport”) AND TOPIC: (“children” OR “adolescents” or “youth” OR “child” OR “teenager”)Refined by: DOCUMENT TYPES: (ARTICLE) AND LANGUAGES: (ENGLISH)Timespan: All years. Indexes: SCI-EXPANDED, SSCI, A&HCI, CPCI-S, CPCI-SSH, ESCI, CCR-EXPANDED, IC.	Article English	126
**Total**			4133
